# Primary prevention of atherosclerosis by pretreatment of low-density lipoprotein receptor knockout mice with sesame oil and its aqueous components

**DOI:** 10.1038/s41598-018-29849-x

**Published:** 2018-08-16

**Authors:** Chandrakala Aluganti Narasimhulu, Kathryn Young Burge, Mitsushita Doomra, Aladdin Riad, Sampath Parthasarathy

**Affiliations:** Burnett School of Biomedical Sciences, College of Medicine, University of Central Florida, Florida, USA

## Abstract

Pharmacological intervention using statins and PCSK9 inhibitors have become first-line therapy in the prevention of hypercholesterolemia and atherosclerosis. Currently, no agent is available for the primary prevention of atherosclerosis. However, there is an emerging hypothesis that atherosclerosis could be driven by inflammation. In this study, we tested whether pretreatment with an aqueous extract from sesame oil (SOAE), which showed potent anti-inflammatory properties without hypocholesterolemic actions, would prevent subsequent atherosclerosis development in a mouse model. RAW 264.7 macrophages and female low-density lipoprotein receptor knockout (LDLR^−/−^) mice were used for *in vitro* and *in vivo* studies, respectively. Plasma lipids, cytokines and atherosclerotic lesions were quantified at the end of the study. RNA was extracted from the liver and aortic tissues and used for gene analysis. Pre-treatment of SOAE prevented Ox-LDL uptake by RAW macrophages and further inflammation *in vitro*. SOAE pre-treatment significantly reduced atherosclerotic lesions and pro-inflammatory gene expressions in LDLR^−/−^ mice as compared to control mice. No significant change in plasma cholesterol levels was observed. A significant reduction in plasma levels of TNF-α, IL-6, MCP-1 and VCAM1 was observed in the SOAE pre-treated animals. This is the first study that demonstrates that pre-treatment with anti-inflammatory agents, could delay/decrease atherosclerosis.

## Introduction

Dyslipidemia and inflammation are associated with atherosclerosis^1–4^. Existing therapeutic approaches for the prevention and treatment of atherosclerosis heavily involve statins or PCSK9 antibodies to reduce hypercholesterolemia^5,6^. Very few, if any, anti-inflammatory agents have been tested for their effectiveness against atherosclerosis.

A cardioprotective diet and exercise have been considered important factors in the prevention and treatment of atherosclerosis. Cooking oil has a great influence on plasma lipid levels as well as on the progression of atherosclerosis^7–10^. Sesame oil (SO) is rich in both polyunsaturated fatty acids (PUFA) and monounsaturated fatty acids (MUFA), and has been shown to reduce high blood pressure and lower the amount of medication required to control hypertension^11,12^ in humans.

Others^13,14^ as well as our studies have demonstrated the ability of SO in reducing plasma lipid levels^15,16^ *in vivo*. In addition, our studies have also revealed that a diet rich in SO prevents inflammation and inhibits atherosclerotic lesion formation (by approximately 85%) in low-density lipoprotein-receptor-deficient (LDL-R^−/−^) male mice^15,16^. This finding led us to evaluate whether non-saponifiable components of edible oil that have been overlooked could have properties that would act in synergy with fatty acid components to not only inhibit atherosclerotic properties, but also promote regression. Subsequently, we demonstrated that an aqueous extract (SOAE) from SO was effective in inhibiting inflammation, both *in vitro* and *in vivo*^17,18^.

Our studies of SO/SOAE diet-fed LDL-R^−/−^ mice showed significantly reduced atherosclerotic lesions as compared to high-fat diet-fed control animals^15,16,18^. The beneficial properties of SO/SOAE prompted us to test whether pre-treatment of SO/SOAE would have anti-inflammatory properties and as a result, decrease subsequent development of atherosclerosis. In addition, we also evaluated whether SO/SOAE pre-treatment would have an effect on lipid metabolism, reverse cholesterol transport (RCT), and scavenger receptors, all of which play a major role in the progression of atherosclerosis.

## Results

### *In vitro* studies

#### Pre-treatment of SOAE reduces Ox-LDL and Ac-LDL uptake by RAW 264.7 cells

As shown in Supplementary Fig. 1, oxidized-low-density lipoprotein/acetylated-low-density lipoprotein (Ox-LDL/Ac-LDL) uptake by macrophages was reduced with increasing concentration of SOAE pre-treatment for 2 hrs as compared to untreated control cells.

#### Pre-treatment of SOAE inhibits Ox-LDL-induced inflammation in RAW 264.7 macrophages

Macrophages were treated with Ox-LDL (25 µg/mL) for 24 h following 2 h pre-treatment by SOAE (50 and 250 µg/mL). RNA was isolated and gene expressions of tumor necrotic factor alpha (TNFα), Interleukin (IL)-1α, IL-1β, IL-6 and monocyte chemoattractant protein 1 (MCP-1) were analyzed. The cells responded well to the positive stimulus of 25 µg/mL of Ox-LDL as seen by significant induction of inflammatory gene expressions. Pre-treatment of SOAE even for 2 hrs significantly inhibited the inflammatory gene expressions in a dose-dependent manner (Supplementary Fig. 2A), whereas a slight but insignificant increase was observed in TNFα at higher concentrations (data not shown). In addition, no significant difference was observed either with lipid loading genes or cholesterol transport genes (data not shown). SOAE treatment without Ox-LDL did not induce any of the inflammatory cytokines. Polymerase chain reaction (PCR) products also corroborated with the gene expression studies (Supplementary Fig. 2B).

### *In vivo* studies

#### Body weight analysis in mice

No significant changes in body/liver weight were observed in the SO/SOAE diet-fed animals, either at one month or following the two additional months of high-fat diet feeding (Supplementary Fig. 3A,B).

#### No significant changes in plasma lipid levels in SO/SOAE diet pre-treated animals

Plasma lipid profile analysis revealed no significant decrease in triglyceride (TRG), total cholesterol (TC), LDL-cholesterol, or very low-density lipoprotein (VLDL)-cholesterol in SO/SOAE diet pre-treated animals either prior to, or after, high-fat diet feeding. However, a slight but insignificant increase in high-density lipoprotein (HDL) cholesterol levels was observed after one month of SO/SOAE feeding in these animals compared to controls. Table 1 represents the plasma lipid profile of control and SO/SOAE diet-fed animals. In control animals, the cholesterol level was 1438.13 ± 132.37 mg/dL, whereas in the animals pre-treated with a SO/SOAE diet, the cholesterol levels were 1343.73 ± 79.8 mg/dL and 1466 ± 94 mg/dL, respectively. No significant change in triglyceride levels was observed. However, there was an increase in HDL levels after one month of feeding of both SO/SOAE and a reduction in TRG levels in SOAE-treated animals.

**Table 1:** Plasma lipid levels in sesame oil diet-fed animals: Plasma lipid levels in mice fed one-month of SO/SOAE diet, followed by two-months of an atherogenic diet.

Plasma lipids	One month treatment			One month followed by 2 month HF diet		
mg/dL	Control	SO	SOAE	Control to HF	SO to HF	SOAE to HF
TRG	97.8 ± 20.9	107.7 ± 22.2	86.8 ± 9.7	247 ± 39.7*	249 ± 39.79*	266.5 ± 26.71*
TC	145.3 ± 15.3	166 ± 19.9	171.8 ± 12.8	1438 ± 132.4*	1343 ± 79.8*	1466.3 ± 94.8*
LDLc	87.3 ± 8	98.4 ± 10.37	102.85 ± 8.72	1310 0.16 ± 128*	1219.16 ± 75.59*	1371.83 ± 94.31*
HDLc	38 ± 4	50.2 ± 7.29	51.71 ± 4.91	72.5 ± 8.19	73.5 ± 6.9	53 ± 3.68
VLDLc	19.56 ± 4.2	21.54 ± 4.44	17.37 ± 1.93	49.5 ± 7.94*	49.96 ± 7.96*	53.3 ± 5.34*
TC/HDL	3.85 ± 0.15	3.37 ± 0.14	3.38 ± 0.12	21.11 ± 2.98	19.16 ± 2.21	42.77 ± 7.2

Values are represented as mean ± SD. *P < 0.05.

However, despite no changes in blood lipids, visual observation of plasma showed a clearer plasma in SO/SOAE diet pre-treated animals as compared to control animals (Supplementary Fig. 4). In lieu of the chemical lipid data, this probably reflected the need, perhaps an association of components of SO/SOAE with the lipoproteins for its actions *in vivo*.

#### Inhibition of atherosclerotic lesion formation in SO/SOAE pre-treated animals

**Figure 1:**
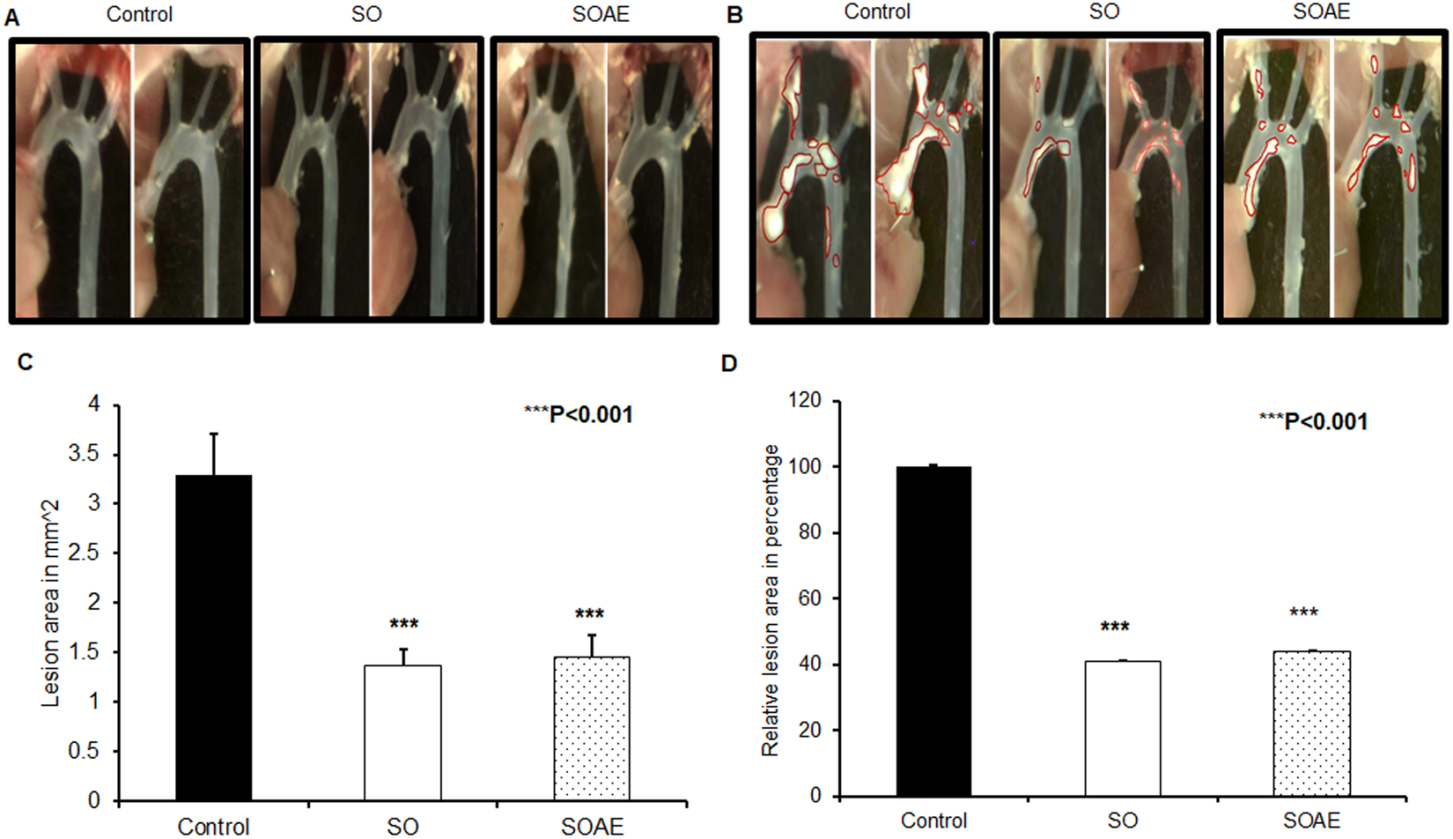
Inhibition of atherosclerosis in SO/SOAE pre-treated animals. Representative images of atherosclerotic lesions in LDLR^−/−^ mice in (**A**) one-month SO/SOAE pre-treated animals; (**B**) one-month SO/SOAE pre-treated followed by two-months high-fat diet animals. (**C**) Average lesion area of all animals from each group; (**D**) Relative (control set to 100) lesion area of animal groups. The values are expressed as mean ± SD (mm^2^). ***p < 0.0001.

Atherosclerotic lesion quantification revealed a significant reduction in lesion area of SO/SOAE pre-treated animals as compared to controls. As represented in Fig. 1A, no lesions were observed in any group at the end of one month of feeding, whereas at the end of two months of feeding, control animals had significant lesion levels in the aortic arch (Fig. 1B) compared to SO/SOAE animals. Quantification analysis also showed a significant (***P < 0.001) reduction in lesion size/area of approximately 55–60% in SO/SOAE animals as compared to controls (Fig. 1C,D: mean ± SD (mm2), ***p < 0.001).

#### Gene analysis of mouse livers

To evaluate the effect of pre-treatment by SO/SOAE, several pro-inflammatory genes (IL-1α, IL-1β, IL-6, MCP-1, myeloperoxidase (MPO) and TNF-α), anti-inflammatory genes (IL-4 and IL-10), lipid-loading genes [cluster differentiation (CD)36 and scavenger receptor (SR) A1], antioxidant genes [(catalase and manganese superoxide dismutase (MnSOD)], genes involved in RCT and lipid metabolism [ATP-binding cassette transporter (ABCA)1, ABCG1, SRB1, Niemann-Pick C1-like protein 1 (NPC1L1), Cytochrome P450 Family 7 subfamily A Member 1 (Cyp7A1)], nuclear receptor transcription factors [liver ×  receptor (LXR) and farnesoid × receptor (FXR)], genes associated with HDL [apolipoprotein A1 (APOA1) and paraoxonase 1 (PON1)], and a matrix metalloproteinase gene (MMP9) were analyzed by real-time PCR analysis (Table 2). The pattern of regulation of pro- and anti-inflammatory genes was consistent with cytokine array analysis. No significant difference was observed in genes involved in RCT and lipid metabolism. Similarly, no difference between pregnane × receptor (PXR) and Peroxisome proliferator-activated receptor-alpha (PPARα) expression was identified. In addition, we also evaluated the Flavin monooxygenase (FMO) 1–5 gene expressions, which play a major role in the formation of trimethylamine N-oxide (TMAO) and disease progression in these animals^19^. Surprisingly, there was a slight but insignificant reduction of these genes in SO/SOAE pre-treated animals.

**Table 2:** Gene analysis from mice liver.

	Control	SO	SOAE	p value	
				Control Vs SO	Control Vs SOAE
**Pro-inflammatory genes**					
IL-1 alpha	1	0.681963	0.504738	0.140	0.820
IL-1beta	1	0.940317	0.451933	0.620	0.980
IL-6	1	0.785231	0.32779	0.490	0.325
MCP-1	1	2.659313	1.504779	0.152	0.170
MPO	1	1.26665	1.658319	0.680	0.650
TNF-alpha	1	0.735319	0.626319	0.330	0.060
**Anti-inflammatory genes**					
IL-4	1	3.321804	0.6711	0.430	0.930
IL-10	1	2.970702	1.393336	0.230	0.640
**Scavenger receptors**					
CD36	1	0.650874	0.426833	0.740	0.360
SRA1	1	0.886013	0.594106	0.580	0.510
**Antioxidant genes**					
Catalase	1	1.413807	1.185208	0.160	0.820
Mn-SOD	1	0.695595	1.301406	0.01*	0.132
**RCT genes**					
APOA1	1	1.885119	1.043672	0.550	0.450
PON1	1	1.943017	1.249621	0.250	0.180
ABCA1	1	3.371703	0.175983	0.900	0.836
ABCG1	1	1.184474	0.949765	0.930	0.790
SRB1	1	0.771615	0.899021	0.228	0.025
NPC1L1	1	0.240529	0.069702	0.450	0.330
**Transcription factors**					
LxR	1	1.192538	0.625569	0.850	0.01*
FxR	1	0.986115	0.423358	0.920	0.280
PxR	1	1.051423	0.774968	0.440	0.500
**Matrix metalloprotease**					
MMP-9	1	0.488572	0.361897	0.290	0.527
**Flavin monooxygenases**					
FMO1	1	0.841205	0.72209	0.820	0.420
FMO2	1	1.180525	0.691214	0.900	0.140
FMO3	1	1.210627	1.086112	0.560	0.180
FMO4	1	0.598995	1.030688	0.070	0.850
FMO5	1	0.886148	1.243417	0.070	0.125

^*^P < 0.05.

#### Gene expression in mouse aorta

**Figure 2:**
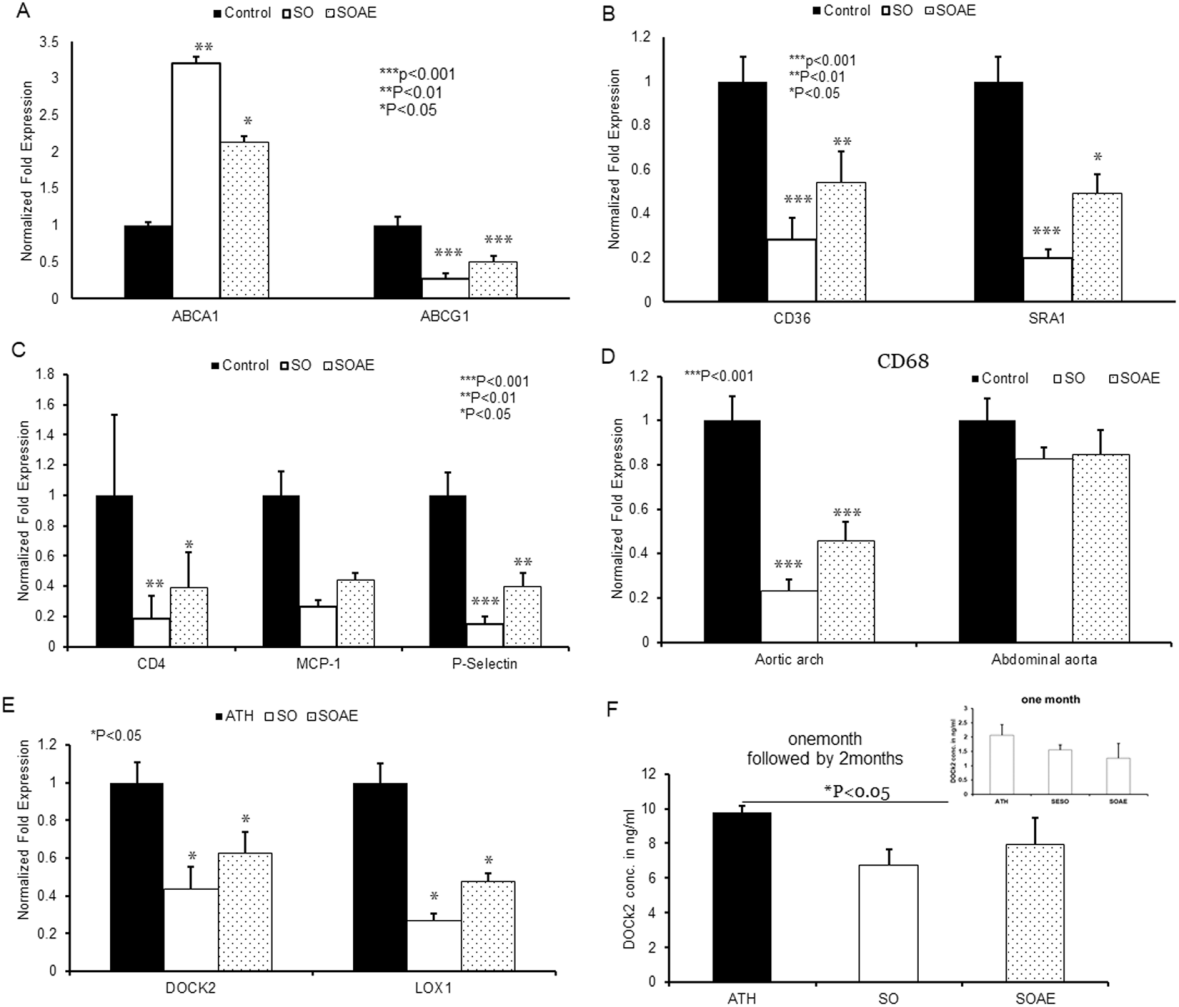
Gene analysis from mouse aorta. mRNA level of several genes were analyzed in aortic tissue of LDLR^−/−^ mice after one month of feeding with SO/SOAE diet, followed by 2 months of feeding with high-fat diet. Bar graph represents (**A**) RCT genes, (**B**) scavenger receptors, and (**C**) inflammatory genes analyzed from the aorta. (**D**) Relative level of CD68 mRNA in aortic arch and abdominal aorta (n = 8 from each group). (**E**) DOCK2 and LOX1 (**F**) DOCK2 ELISA. Values are represented as mean ± SD. *p < 0.05.

Aortic gene expressions were also analyzed using real-time PCR. No difference was observed in one-month SO/SOAE pre-treated animals as compared to control animals. However, following the two-month high-fat feeding, the results showed a significant increase in RCT ABCA1 gene expression and significantly reduced levels of ABCG1 expression in SO/SOAE pre-treated animals (Fig. 2A). Surprisingly, one-month pre-treatment with SO/SOAE significantly reduced the expression of monocyte/macrophage markers and scavenger receptors SRA1 and CD36 (Fig. 2B). In addition, a significant reduction was observed in inflammatory markers such as MCP-1, CD4 and P-selectin (Fig. 2C) in experimental animals as compared to control animals. A significant difference in the level of CD68 between control and experimental animals in the aortic arch segments was observed, whereas no such difference was observed in lesion-free abdominal aorta segments (Fig. 2D). SO/SOAE pre-treatment decreased CD68 levels in the aortic arch by 80% and 60% (p < 0.05), respectively. Dedicator of cytokinesis 2 (DOCK2) and lectin-like oxidized low-density lipoprotein (LOX1) also significantly reduced in SO/SOAE pre-treated animals (Fig. 2E). Protein levels by ELISA for DOCK2 also corroborated the gene expression studies (Fig. 2F). As shown in Fig. 2F, a significant reduction in plasma DOCK2 levels was observed in SO pre-treated animals, whereas a non-significant reduction was observed in SOAE pre-treated animals.

#### Gene expression in mouse peritoneal macrophages

**Figure 3:**
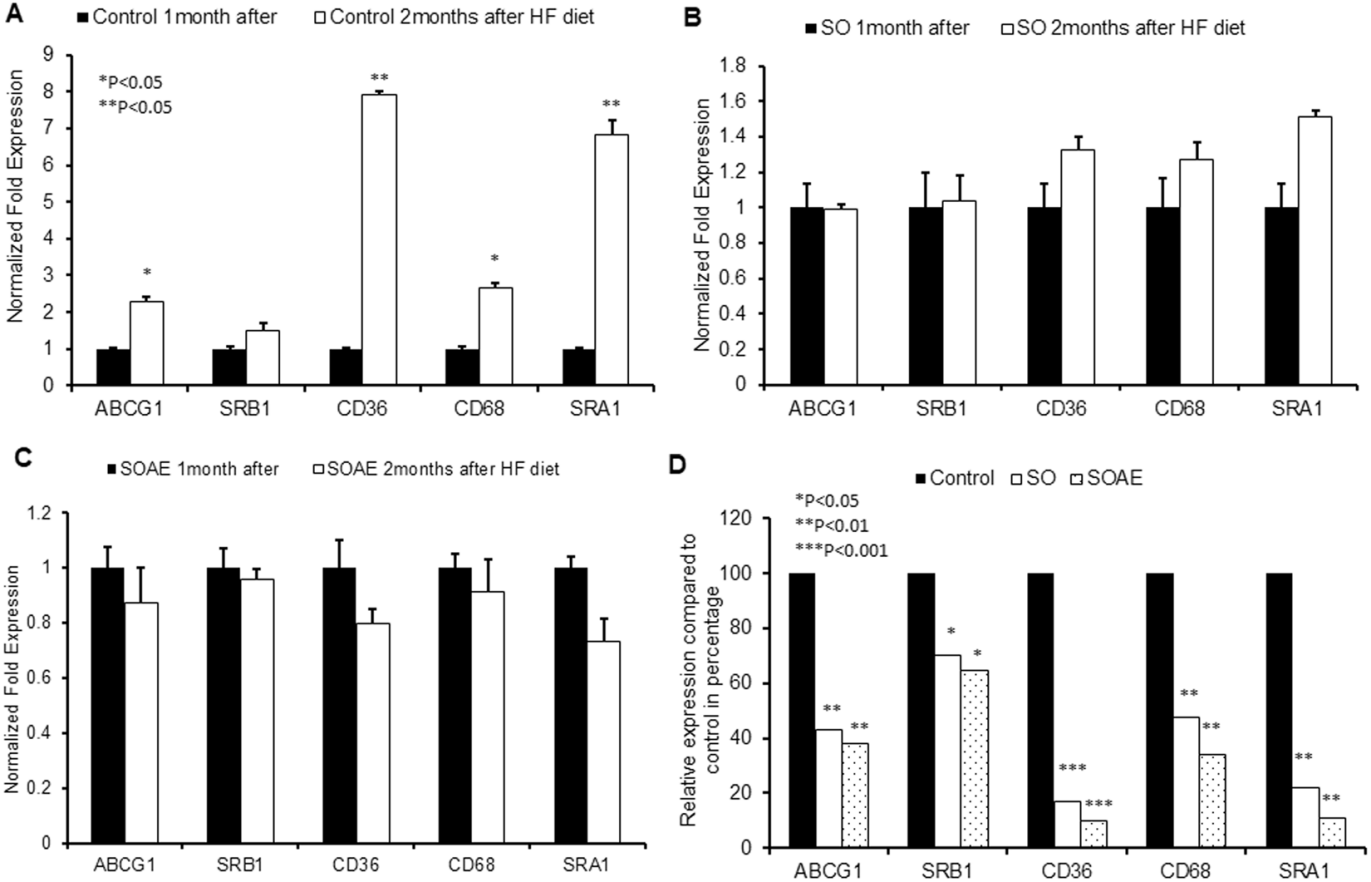
Gene analysis from mice peritoneal macrophages. mRNA level of several genes were analyzed in peritoneal macrophages of LDLR^−/−^ mice one month after SO/SOAE diet and followed by 2 months of feeding with high-fat diet. Bar diagrams represent RCT genes and Scavenger receptors (**A**) Control one month diet and followed by 2months HF diet (**B**) SO one month diet and followed by 2months HF diet (**C**) SOAE one month diet followed by 2months HF diet (**D**) Relative increase/decrease with respect to control in SO and SOAE pre-treated animals were analyzed from peritoneal macrophages (n = 8 from each group). Values are represented as mean ± SD. *P < 0.05.

Peritoneal macrophage gene expressions were also analyzed in addition to the liver and aortic gene expressions, using real-time PCR. The results showed that SO/SOAE pre-treated animals had reduced levels of cholesterol transport gene expressions (ABCG1, SRB1, SRA1 and CD36) compared to control animals (Fig. 3A–C). A significant reduction (40–60%) in cholesterol uptake gene expression was observed in SO/SOAE animals, suggesting even the short-term pre-treatment of SO/SOAE may play a beneficial role in maintaining cholesterol homeostasis. Additionally, there was a significant increase in all cholesterol transport gene expressions at the end of the one-month treatment, suggesting accelerated lipid metabolism in the presence of SO/SOAE.

#### Cytokine array

Cytokine array revealed that SO/SOAE pre-treatment induced minimal to significant changes, depending on the protein. Upregulation of growth-promoting cytokines was observed in SO/SOAE pre-treated animals as compared to controls, whereas several pro-inflammatory cytokines such as granulocyte-macrophage colony-stimulating factor (GMCSF), IL-6, MCP-1, TNF-α, TNFRI, TNFRII, and vascular cell adhesion molecule 1 (VCAM1) were significantly downregulated (Table 3). Anti-inflammatory cytokines, such as IL4 and IL-10, were upregulated immediately following the one-month SO/SOAE pre-treatment (data not shown).

**Table 3:** Cytokine array: Comparison of changes in the level of inflammatory mediators in mouse plasma between pre-treated SO/SOAE animals and controls.

Protein name	Protein symbol	Relative protein levels compared to ATH, fold difference			P value C vs SO	P value C vs SOAE
		Control	SO	SOAE		
Tyrosine-protein kinase receptor	axl	1	0.4149	0.4267	0.0124*	0.0262*
B cell Attracting Chemokine-1	BLC	1	0.4791	0.4336	0.0019*	0.0610
CD30 ligand	CD30L	1	0.5604	0.6153	0.0453*	0.0052*
Cluster of differentiation 30	CD30/TNFRSF8	1	0.5650	0.7813	0.9839	0.1208
Cluster of differentiation 40	CD40	1	0.6379	0.8003	0.5543	0.1949
Cytokine responsive gene 2	CRG-2	1	0.8479	0.7188	0.3718	0.6064
Cuteaneous T-Cell Attracting Chemokine	CTACK	1	0.8372	0.7293	0.2280	0.5573
Chemokine 16	CXCL16	1	0.3503	0.4235	0.4722	0.3235
E-Selectin	E-Selectin	1	0.9837	0.9848	0.8765	0.8410
Low affinity immunoglobulin gamma Fc region receptor II-b	Fcg RIIB	1	0.5797	0.8134	0.0741	0.3747
Fractalkine	Fractalkine	1	0.4488	0.5791	0.7109	0.7708
Granulocyte colony stimulating factor	G-CSF	1	0.9592	0.8227	0.8315	0.8108
Granulocyte macrophage colony stimulating factor	GM-CSF	1	0.6072	0.7116	0.0199*	0.0226*
Hepatocyte growth factor receptor	HGF R	1	0.8084	0.9227	0.5090	0.7133
Intracellular adhesion molecule 1	ICAM-1	1	0.7731	0.7365	0.2928	0.1603
Interferon gamma	IFN-gamma	1	0.4943	0.6302	0.0201	0.1071
Insulin-growth factor binding protein 2	IGFBP-2	1	0.9519	0.9933	0.7459	0.9591
Insulin-growth factor binding protein 3	IGF-BP-3	1	0.6091	0.6441	0.3863	0.2837
Insulin-growth factor binding protein 5	IGF-BP-5	1	0.8475	0.8614	0.3754	0.2825
Inteleukin 2	IL2	1	0.5927	0.5730	0.0129*	0.0473*
Interleukin 3	IL3	1	0.6250	0.7643	0.0014*	0.0547
Interleukin 3 receptor B	IL3 Rb	1	0.7283	0.7316	0.5470	0.4356
Interleukin 5	IL5	1	0.5039	0.7117	0.0159	0.0063*
Interleukin 6	IL6	1	0.5311	0.6715	0.0390*	0.0153*
Interleukin 9	IL9	1	0.4989	0.5826	0.0079*	0.0029*
Interleukin 12 P40/P70 subunits	IL12-p40/p70	1	0.4908	0.8024	0.6243	0.0579
Interleukin 12 P70 subunit	IL12-p70	1	0.6393	0.6336	0.0216*	0.0744
Interleukin 17	IL17	1	0.5014	0.8513	0.0154*	0.4477
Leptin receptor	Leptin R	1	0.6288	0.4419	0.0180*	0.0813
Leptin receptor	Leptin	1	0.4129	0.8535	0.3866	0.5763
Lymphocyte selectin	L-Selectin	1	0.8507	0.6533	0.0157*	0.0001*
Lymphotactin	Lymphotactin	1	0.6955	0.6483	0.2066	0.0233*
Monocyte chemotactic protein 1	MCP-1	1	0.4663	0.5434	0.0037*	0.0102*
Monocyte chemotactic protein 5	MCP-5	1	0.6026	0.6870	0.7321	0.7859
Monokine induced by gamma interferon	MIG	1	0.3939	0.8645	0.0001*	0.4457
Macrophage inflammatory protein 1 alpha	MIP-1-alpha	1	0.9069	0.9083	0.9007	0.8884
Macrophage inflammatory protein 3 beta	MIP-3-beta	1	0.9786	0.8468	0.2460	0.6249
Macrophage inflammatory protein 3 alpha	MIP-3-alpha	1	0.7446	0.8197	0.2937	0.3140
Matrix metalloproteinase 3	MMP-3	1	0.9984	0.9609	0.9915	0.7725
Platelet factor 4	PF4	1	0.9058	0.8626	0.9482	0.3180
Platelet selectin	P-Selectin	1	0.7903	0.5439	0.9901	0.2998
Resistin	Resistin	1	0.9654	0.9619	0.4734	0.1207
Stem cell factor	SCF	1	0.8123	0.9165	0.0910*	0.0171*
Stromal cell derived factor 1 alpha	SDF-1 alpha	1	0.6650	0.7799	0.0014*	0.0001*
Sonic hedgehog-N terminal domain	Shh-N	1	0.9726	0.9027	0.9206	0.7007
Thymus and activation-regulated chemokine	TARC	1	0.5413	0.9009	0.0081*	0.0607
T Lymphocyte-Secreted Protein	TCA-3	1	0.9283	0.9559	0.0707	0.4547
Thymus-Expressed Chemokine	TECK	1	0.4709	0.5847	0.0708	0.0535
Tissue inhibitor of metalloproteinase-1	TIMP-1	1	0.9769	0.9920	0.7220	0.6062
Tumor necrotic factor-alpha	TNF-alpha	1	0.5126	0.5730	0.0093*	0.0470*
TNF-related activation-induced cytokine	TRANCE	1	0.9140	0.9003	0.5615	0.5172
Soluble tumor necrotic factor receptor 1	sTNF RI	1	0.9708	0.7883	0.0008*	0.0018*
Soluble tumor necrotic factor receptor 2	sTNF RII	1	0.1775	0.1892	0.0001*	0.0001*
Thrombopoetin	TPO	1	0.7166	0.6588	0.0244*	0.0334*
Vascular cell adhesion molecule 1	VCAM-1	1	1.0756	0.7386	0.2952	0.0015*
Vascular endothelial growth factor	VEGF	1	0.4647	0.2821	0.0019*	0.3102

Three samples from each group were analyzed by Ray-Bio® cytokine array analysis. The protein levels are expressed as fold-change between the groups. ^*^P < 0.05.

## Discussion

In the present study, we observed that pre-treatment with a SO/SOAE-supplemented diet effectively reduced subsequent atherosclerotic lesion formation in LDLR^−/−^ female mice. No change in the plasma levels of TC, TRG, VLDLc, HDLc and LDLc was observed in these pre-treated animals as compared to atherosclerotic diet (control) animals. In our earlier studies with azelaic acid, we noted similar inhibition of atherosclerosis without appreciable changes in plasma lipid levels^20^. Based on the decreased number of CD68 positive macrophages in the atherosclerotic artery, we concluded that recruitment of leukocytes and reduced inflammation could have played a significant role in SO/SOAE’s mode of action, as opposed to lipid-lowering. A number of studies have demonstrated that knockdown of specific inflammatory mediator(s) affect atherosclerosis^21,22^. However, compensatory mechanisms and multiple interactions of inflammatory pathways could have affected the outcome. Our studies provide direct evidence for a role of pro-inflammatory cytokines in atherosclerosis and an alternative role for total or LDL-associated cholesterol in atherogenesis. We would like to exercise caution in the interpretation of our data, as lipids were increased in the high fat-fed state and the study did not measure changes in lipid classes or their distribution amongst subfractions. The study also did not measure specific pro- or anti-inflammatory proteins that could be associated with the lipoprotein classes.

Reduced mRNA levels of pro-inflammatory cytokine genes and scavenger receptor genes (CD36 and SRA1) were observed in SO/SOAE pre-treated animals liver tissue. Increased expression of antioxidant genes (catalase) and anti-inflammatory cytokine genes (IL-4 and IL-10) was also observed, suggesting that the pre-treatment had a significant influence on not only modulating cholesterol homeostasis but also upon lipid-loading and the generation of inflammation.

Enhanced RCT with increased expression of ABCA1 in SO/SOAE pre-treated animals was observed as compared to control animals. The increased ABCA1 and reduced ABCG1 in SOAE pre-treated animals might suggest that SOAE might promote enhanced RCT towards lipid-poor APOA1 rather than to intact HDL. This might be also due to the altered cytoskeleton which might promote increased binding of free APOA1 to the macrophage surface. These are mere speculations and need to be experimentally verified. It is to be pointed out that no significant changes in HDL were noted with SOAE; however, free and bound APOA1 were not measured. In contrast to ABC A1, ABC G1 appears to be promiscuous in its transport properties and it also appears to deliver cholesterol to LDL. Considering its complex actions, more studies are needed. No significant changes were observed in nuclear receptors such as LXR, FXR and Retinoid X receptor (RXR) in SO/SOAE pre-treated animals as compared to control animals.

Increased PON1 and APOA1 in SO/SOAE pre-treated animals suggests enhanced functionality of HDL as well as protection against oxidative stress-induced atherosclerosis. PON1 is synthesized in the liver and is thought to be exclusively associated with HDL^23–26^.

An insignificant reduction in MMP9 gene expression was observed in SO/SOAE pre-treated animals. MMP9 has been considered a major pathological factor in atherosclerosis due to its significant role in migration and proliferation of smooth muscle cells during plaque formation. Further, reduced plasma levels of MMP3 in SO/SOAE pre-treated animals suggest that one-month pre-treatment is quite effective in reducing matrix metalloproteinases. Evidence suggests that DOCK2 plays a major role in the phenotype modulation, migration and proliferation of smooth muscle cells (SMCs), a major event in lesion formation and in progression of atherosclerosis^27–31^. Further, these studies also suggest that targeting DOCK2 would be the best therapeutic approach to control disease progression. In our studies, we observed a significant reduction in DOCK2 expression in the aorta of SO/SOAE pre-treated animals, suggesting and confirming the ability of SO/SOAE in targeting DOCK2 by dietary approach.

A significant reduction in mRNA levels of CD36 and SRA1 was observed in aortic lesions of SO/SOAE pre-treated animals, suggesting the ability of both SO and its aqueous component (SOAE) to prevent foam cell formation. SO contains lignans such as sesamin, sesamolin and asarinin, which play an important role in promoting health^32,33^. In addition, sesamin and sesamolin have already shown antioxidant^34^, antiproliferative^35,36^, antihypertensive^37,38^, and neuroprotective activities^39^, as well as the ability to lower cholesterol levels^40^ and increase hepatic fatty acid oxidation enzymes^41^. Lignans of SO are known to form complexes with cholesterol and further prevent the latter’s absorption^42,43^. These lignans might also influence the reduced plasma lipids in SO treated animals^42^. The results of our studies also suggest the absence of these lignans in SOAE.

We also observed a decrease in the mRNA level of CD68 a monocyte/macrophage marker in SO/SOAE pre-treated animals as compared to controls. This decrease might suggest and confirms the reduced presence of foam cell-forming macrophages in SO/SOAE pre-treated animals atherosclerotic lesions (Fig. 2). Similarly, a significant reduction in levels of MCP-1 and CD4 was observed in SO/SOAE pre-treated animals as compared to control animals (Fig. 2), suggesting the possibility of reduction in additional chemotactic factors which might have contributed to the reduced occurrence of macrophages. The diminished levels of CD68 mRNA was also observed in the abdominal aortic segment, which did not contain any visible lesions.

Evidence suggests the upregulation of LOX-1 receptor under pro-atherogenic conditions^44–49^, and its presence in atherosclerotic lesions of both humans and animals^44,48^. According to studies from Mehta and Thakkar *et al*.^50,51^, LOX1 is a key player in the progression of atherosclerosis and targeting the LOX1 might be an effective therapeutic strategy. In our studies, both SO and SOAE pre-treatment significantly reduced the LOX1 expression in aortic lesions, suggesting its role in preventing the internalization of Ox-LDL followed by inflammation initiation, an early event which leads to a cascade of actions in disease progression.

Our *in vitro* studies suggest the pre-treatment of SOAE for 2 hr is able to inhibit the Ox-LDL-induced inflammation in RAW cells; similarly, *in vivo* studies also suggest the short-term pre-treatment is able to reduce inflammation and oxidative stress even in the presence of a high fat diet.

Our data suggest that SO/SOAE pre-treatment is able to inhibit subsequent development of atherosclerosis. To our knowledge, this is the first evidence that, despite no drastic changes in plasma lipid levels, atherosclerosis could be prevented and controlled by the manipulation of leukocyte recruitment and by reducing their inflammatory responses. These results prompted us to analyze the components of SOAE by LC-MS, select specific components, and further test their role against inflammation. Indeed, the components of SOAE in combination counteracted against LPS-induced inflammation in monocyte-derived macrophages^52^. Considering the fact that the lipophilic molecules of SO, such as lignans, α–tocopherol and phytosterols, have less feasibility to separate out into water (confirmed by the absence of these in SOAE by LC-MS analysis), our published studies^17,18^ suggest that apart from these lipophilic molecules, SO has other biologically active polar molecules that can counteract inflammation and atherosclerosis. However, the study should in no way be interpreted to suggest that ALL the inflammatory components reside in the aqueous extract. An aqueous extract is a simple extract and could be prepared and delivered easily. It could be concentrated and given as a pill or as a capsule. Sesame oil, despite its anti-inflammatory properties, is still an oil and contains huge amounts of lipids. A human dose of 30 ml is roughly over 250 calories. In a dyslipidemic situation, it would be undesirable to consume large quantities of fat.

Considering the plant-derived pharmaceutical nature of the extract, SOAE and related agents could be used in young and vulnerable subjects without long-term commitment to statins or other drugs. The combinational approach of SOAE and statins are our ongoing as well as future studies, as statins are potent lipid-lowering drugs.

## Methods

### Chemicals

All PCR primers and Trizol^TM^ reagent were purchased from Invitrogen (Carlsbad, CA). DOCK2 Elisa kit was purchased from My Biosource (MBS9328886: San Diego, CA).

### Cell culture

RAW 264.7 mouse macrophages (ATCC, Manassas, VA) were cultured in RPMI-1640 medium supplemented with 10% fetal bovine serum (FBS).

### Isolation and oxidation of lipoproteins

LDL was isolated from normal plasma by sequential ultracentrifugation using a Beckman TL-100 tabletop ultracentrifuge (Beckman, Palo Alto, CA)^53,54^. LDL was then dialyzed and filter-sterilized. Following estimation of protein concentration, LDL samples were subjected to oxidation with 5 µM copper in 1 ml of phosphate-buffered saline (PBS) at 37 °C. The formation of conjugated dienes was monitored at an optical density of 234 nm for about 18 h using SLM Amnico DB-3500 spectrophotometer (Urbana, IL). The degree of LDL oxidation was assessed by determination of peroxide content using leucomethylene blue (LMB) and thiobarbituric acid reactive substances (TBARS) assay. LDL was acetylated (Ac-LDL) using acetic anhydride. Electrophoretic mobility of modified lipoproteins was determined by agarose gel electrophoresis (Supplementary Fig. 5).

### Preparation and analysis of SOAE

SOAE was prepared and used as described previously^17^.

### Analysis of Ox-LDL/Ac-LDL uptake

RAW 264.7 cells were pre-treated with SOAE for 2 h followed by incubation with Ox-LDL (25 µg/ml) or Ac-LDL (10 µg/ml) for 24 h. Cells were stained with Oil Red O and images were recorded. To monitor the changes in genes involved in inflammation as well as in RCT, cells were harvested in Trizol™ for RNA isolation after 24 h of incubation.

### Animals

Fifty, 4-week-old female LDLR^−/−^ mice weighing 18–20 g were obtained from Jackson Laboratory (Bar Harbor, ME). After 2-weeks of acclimatization, six animals were sacrificed to obtain baseline parameters. The remaining animals were divided into three groups: group 1 was fed a normal diet (n = 14), group 2 (n = 15) was fed sesame oil reformulated a normal diet, and group 3 (n = 15) was fed an SOAE-supplemented normal diet for 1 month. After one month, 6, 7 and 7 animals were sacrificed from each group, respectively. The remaining animals were fed an atherogenic diet for 2 months following the pre-treatment period. The animals were regularly monitored, and a weekly record of body weight was maintained up to 90 days. All procedures were performed according to the protocol approved by The Institutional Animal Care and Use Committee of the University of Central Florida and all methods were performed in accordance with the relevant guidelines and regulations.

### Diet

The normal Purina diet (TD.150278) with 1% soybean oil, normal diet reformulated with either 1% SO (TD.150515) or SOAE (prepared in-house) and an atherogenic diet with 0.2% cholesterol and 17% high fat (TD.04287) were purchased from Harlan Teklad (Madison, WI). Diets were stored at 4 °C to avoid oxidation.

### Isolation of mouse peritoneal macrophages

Macrophages from the peritoneal cavity of all groups of animals were isolated by peritoneal lavage using 3 ml of cold saline and centrifugation at 1200 rpm for 5 min. Cells were utilized for RNA isolation.

### Collection of plasma and organs

After 4 and 12 weeks, mice were fasted overnight and anesthetized with 1–2% isoflurane. Fasting blood samples were collected into EDTA tubes by heart puncture. Plasma was separated as described previously^15^ and stored at −80 °C. The liver was perfused with PBS, weighed, and the tissue used for RNA isolation.

### Isolation and quantification of aortic lesions

Isolation of the aorta and quantification of aortic lesions was performed as described previously^15,16,18^. Lesion areas were marked on photographs. The lesion area was quantified using ImageJ software^55^. After imaging, aortas were saved for RNA extraction.

### Plasma lipid analysis

Plasma lipid profiles of total cholesterol (TC), triglyceride (TRG), HDL cholesterol (HDLc), and LDL cholesterol (LDLc) were determined using a Cholestech L*D*X analyzer (Cholestech Corp, Hayward, CA).

### cDNA synthesis and RT-PCR reaction

Total RNA from cells, liver and aortic tissue was isolated by using Trizol^TM^ reagent. Total RNA of 1 µg was then reverse-transcribed into cDNA using the Superscript^TM^ III First Strand Synthesis system (Invitrogen, Carlsbad, CA). cDNA (50 ng) samples were used to perform Quantitative real-time PCR by iQTM5 iCycler Multicolor Real-Time PCR Detection System (Bio-Rad, Hercules, CA) with SYBR Green (Invitrogen, Carlsbad, CA). Mouse oligonucleotide primers for RT-PCR were purchased from Invitrogen (Carlsbad, CA). Polymerase chain reaction (PCR) was carried out with ABCA1, ABCG1, SRB1, Cyp7a1, FMO1-5, NPC1L1, MCP-1, IL-1α, IL-1β, IL-6, IL-4, IL-10, IL-13, catalase, MnSOD, SRA1, CD36, LXR, PXR, FXR, MPO, CD68, DOCK2 and LOX1 with mouse-specific primers (Supplementary Table 1), resulting in a 200 bp fragment each for sample. As a reference gene, GAPDH primers were utilized. PCR was performed with an initial step of denaturation at 50 °C for 2 mins, 95 °C for 10 mins followed by 40 cycles of 95 °C for 20 s and 60 °C for 20 s. Melt curves were established for the reactions. Normalized fold expression was calculated by using 2^−∆∆Ct^ method.

The entire reaction products were electrophoresed on 2% agarose gels.

### Global Cytokine array

Plasma samples were analyzed by the global cytokine array by Ray Biotech Inc. (Norcross, GA) using RayBio^®^Mouse G Series Array 3 and 4 glass chip.

### DOCK 2 ELISA

Fifty microliters of samples were analyzed using a sandwich ELISA kit (My Biosource Inc, CA) following manufacturer’s protocol. The absorbance was measured at 450 nm using a microplate reader (Bio-Rad, Hercules, CA). Concentration of DOCK2 was expressed in ng/ml.

### Statistics

Values are presented as means ± standard deviation (SD), and statistical analyses were performed by using student’s t-test, with *p* < 0.05 as the level of significance. Significance between groups was calculated by using two-tailed student’s t-test and Wilcoxon matched paired test using Prism Pad software (version V), with *p* < 0.05 considered significant.

## Electronic supplementary material

Supplemental material
