# Supplementary material for: Primary prevention of atherosclerosis by pretreatment of low-density lipoprotein receptor knockout mice with sesame oil and its aqueous components
Source: Sci Rep. 2018 Aug 16;8:12270. doi: 10.1038/s41598-018-29849-x (PMC6095901; doi:10.1038/s41598-018-29849-x)
Supplement: Supplementary file 1 — Supplemental material [file 41598_2018_29849_MOESM1_ESM.docx]

**Title**: Primary prevention of atherosclerosis by pretreatment of low-density lipoprotein receptor knockout mice with sesame oil and its aqueous components

**Author(s):** Chandrakala Aluganti Narasimhulu, Kathryn Young Burge, Mitsushita Doomra, Aladdin Riad, and Sampath Parthasarathy*

Supplementary Figure. 1: SOAE reduces Ox-LDL and Ac-LDL uptake by RAW 264.7 cells


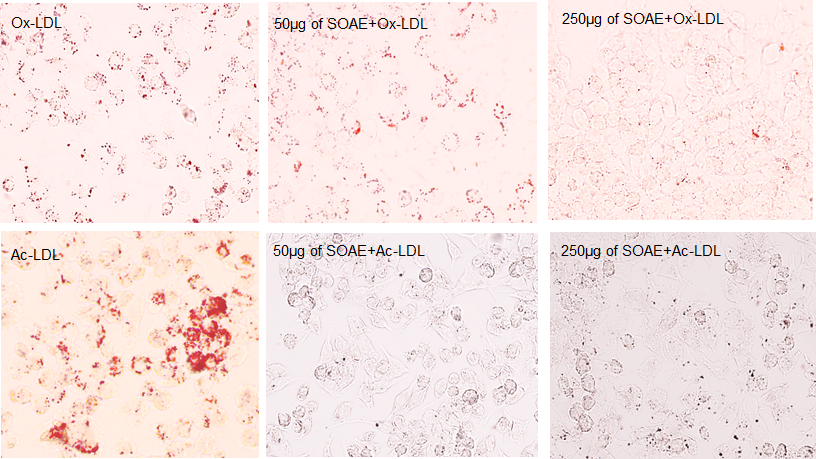


Supplementary Figure 2: SOAE pre-treatment inhibits Ox-LDL induced inflammation in RAW 264.7 macrophages. A)Gene expression B) PCR products i)GAPDH ii)IL-1 alpha iii)IL-1 beta

iv)IL-6 v)MCP-1.


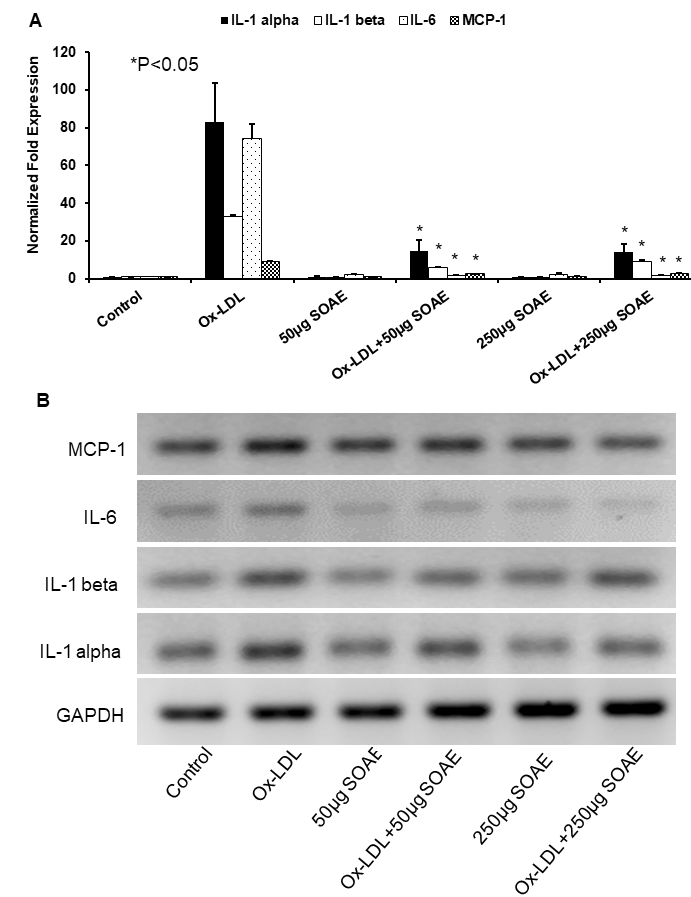


**B**


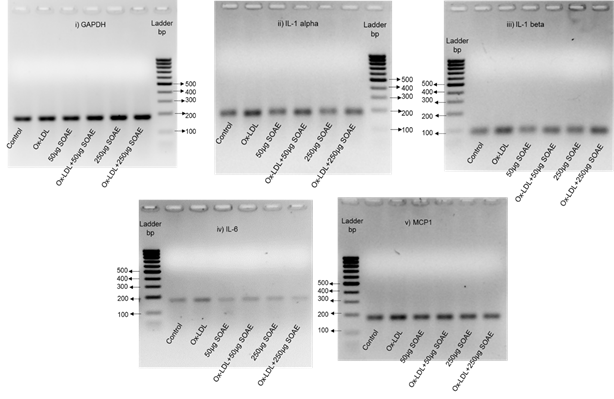


Supplementary Figure. 3: Body and liver weight of mice


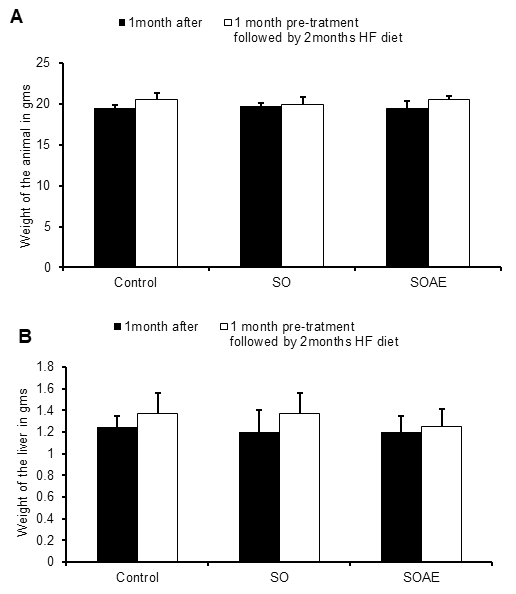


Supplementary Figure.4: Plasma samples of animals


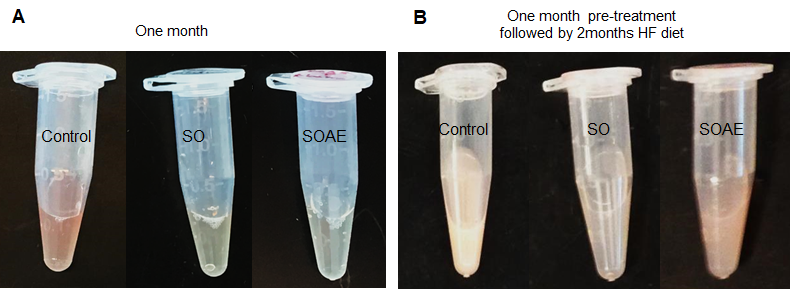


Supplementary Figure. 5: Lipoproteins were isolated from the plasma of consented subjects and used for oxidation with copper. 100 μg LDL was oxidized with 5 μM copper in 1 ml PBS and OD was measured at 234 nm (A). Electrophoretic mobility of modified lipoproteins was determined by Agarose gel electrophoresis (B). Peroxide formation Ox-LDL was confirmed by LMB (C) and TBARS (D) assays.


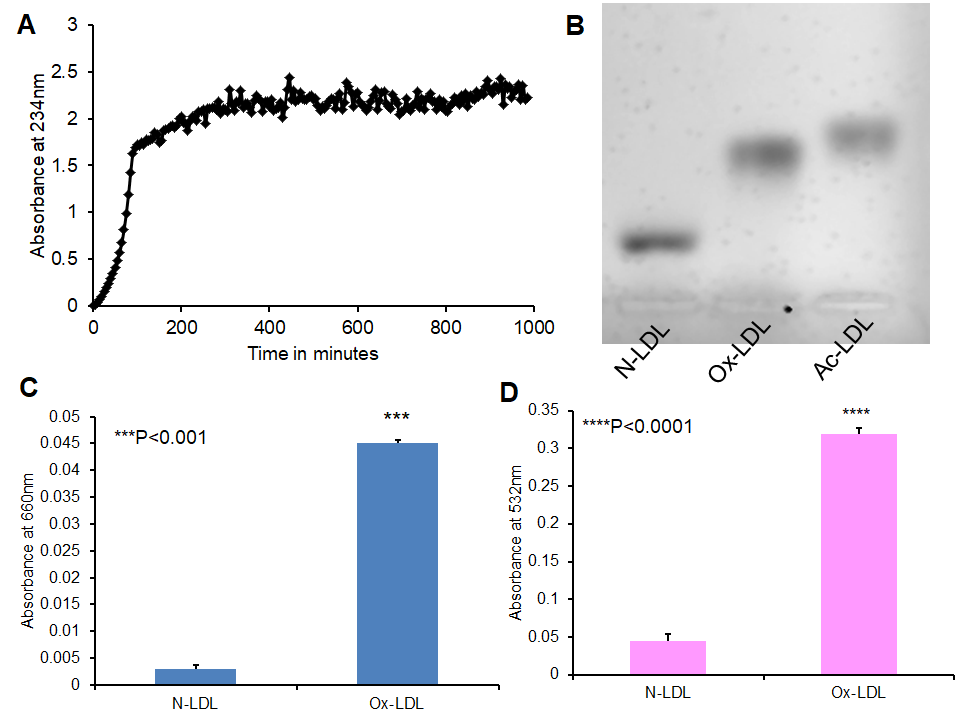


Supplementary Table 1: List of oligonucleotide primers used for RT-PCR

| **Target** | **Forward Primer** | **Reverse Primer** |
| --- | --- | --- |
| ABCA1 | 5'-GGGAATTGAACCTGAGTCCT-3' | 5'-AGTCATTCCTCCTCCCATTC-3' |
| ABCG1 | 5'-CCAGACAGTTGTGGATGTGG-3' | 5'-GACCTCGCTCTTCCTTCCTT-3' |
| APOA1 | 5'-CCAATGGGACAAGTGAAGG-3' | 5'-ACGGTTGAACCCAGAGTGTC-3' |
| Catalase | 5'-ACGCTTCAACAGTGCTAATG-3' | 5'-GTTCTCACAGGGGTTTC-3' |
| CD4 | 5’-GACCCTGAAGCAGGAGAACC-3’ | 5’-TGCCTGGCGCTGTTGG-3’ |
| CD36 | 5'-TGCTGGAGCTGTTATTGGTG-3' | 5'-TGGGTTTTGCACATCAAAGA-3' |
| CD68 | 5'-TAGCCCAAGGAACAGAGGAA-3' | 5'-TGGCAGGGTTATGAGTGACA-3' |
| CYP7A1 | 5'-ACATGGAGAAGGCTAAGACG-3' | 5'-CTTCTTCAGAGGCTGCTTTC-3' |
| DOCK2 | 5’-GGTTGGATGAGGCCAACACA-3’ | 5’-ACTTGCTAGCCAGCATGGTC-3’ |
| FMO1 | 5’-CTCGTGCCGCTGGGAT-3’ | 5’-GTGGAGGGAGCTGTGTTCTG-3' |
| FMO2 | 5’-GCCTACTGGAGTTACCTGACC-3' | 5’-TCAGTCCTTTCGAAGCAGGT-3' |
| FMO3 | 5’-ATTCGGCACGAGAAAGGAA-3' | 5'-CCTGCCCTCTTCTATATGGTCT-3' |
| FMO4 | 5’-ACGGGACAGGGCAGTATTTG-3' | 5'-CTATGTCCCCGCCAGTGTTT-3' |
| FMO5 | 5’-CCACAGAGAAAGCCCAGACA-3' | 5'-AGCAGCAGGCACAAGTAGAG-3' |
| FXR | 5'-ACATCCCCATCTCTCTGC-3' | 5'-TGTGAGGGCTGCAAAGGTT-3' |
| IL-1α | 5'-GCAACGGGAAGATTCTGAAG-3' | 5'-TGACAAACTTCTGCCTGACG-3' |
| IL-1β | 5'-AACCTGCTGGTGTGTGACTTC-3' | 5' -CAGCACGAGGCTTTTTTGT-3' |
| IL-4 | 5'-GCGACAAAAATCACTTGAGAG-3' | 5'-CCTTGGAAGCCCTACAGAC-3' |
| IL-6 | 5'-AGTTGCCTTCTTGGGACTGA-3' | 5'-TCCACGATTTCCCAGAGAAC-3' |
| IL-10 | 5'-AACCTGCTGGTGTGATTC-3' | 5'-CAGCACGAGGCTTTTTTGT-3' |
| LOX1 | 5’-CATCCTCTGCCTGGTGTTGT-3’ | 5’- TCCTGCTGAGTAAGGTTCGC-3’ |
| LXR | 5'-ACCTCTGCAATCGAGGTC-3' | 5'-GGTTGATGAACTCCACCTG-3' |
| MCP-1 | 5'-CAGCAAGATGATCCCAATGA-3' | 5'-TGGTTCCGATCCAGGTTTT-3' |
| MMP-9 | 5'-CGACGACGACGAGTTGTG-3' | 5'-CTGTGGTGCAGGCCGAATAG-3' |
| MnSOD | 5'-CTAAGGGTGGTGGAGAACC-3' | 5'-ACCTTGGACTCCCACAGAC-3' |
| MPO | 5'-CCACTCCTGCCAAAACTGAAT-3' | 5'-CACTGTGCTAGGCTGTGGAA-3' |
| NPC1L1 | 5'-GTCTGTCCCCGCCTATACAAA-3' | 5'-GGTCAGGGCTGCAGGTATTA-3' |
| PON1 | 5'-AGAGGTGCTTCGAATCCAGA-3' | 5'-AACACTGTGCCAATCAGCAG-3' |
| P-Selectin | 5’-AACCACTGCCAACCTGTGAA-3’ | 5’-GGCTTTACTGGGAACCGGAA-3’ |
| PXR | 5'-GACCTGCCTATTGAGGACCA-3' | 5'-TTCTGGAAGCCACGATTAGG-3' |
| SRA1 | 5'-AAAGGTGATCGGGGACAAA-3' | 5'-TTGCCCCAATATGATCAGG-3' |
| SRB1 | 5'-GGGCTCGATATTGATGGAGA-3' | 5'-GGAAGCATGTCTGGGAGGTA-3' |
| TNF-α | 5'-CACACTCAGATCATCTTCCAAAA-3' | 5'-GCAATGACTCTAAGTAGACCTGC-3' |
| PPARα | 5'-AAGAGGGCTGAGCGTAGGT-3' | 5'-GGCCGGTTAAGACCAGACT-3' |
